# Reconstruction of composite defects of the scalp and neurocranium—a treatment algorithm from local flaps to combined AV loop free flap reconstruction

**DOI:** 10.1186/s12957-018-1517-0

**Published:** 2018-11-07

**Authors:** Dominik Steiner, Raymund E. Horch, Ilker Eyüpoglu, Michael Buchfelder, Andreas Arkudas, Marweh Schmitz, Ingo Ludolph, Justus P. Beier, Anja M. Boos

**Affiliations:** 1 Department of Plastic and Hand Surgery, University Hospital of Erlangen, Friedrich-Alexander University of Erlangen-Nuernberg, Krankenhausstr. 12, 91054 Erlangen, Germany; 2 Department of Neurosurgery, University Hospital of Erlangen, Friedrich-Alexander University of Erlangen-Nuernberg, Erlangen, Germany; 3 Department of Plastic Surgery, Hand and Burn Surgery, University Hospital RWTH Aachen, RWTH, Aachen, Germany

**Keywords:** Interdisciplinary reconstruction, Composite defects of the neurocranium, Free flap coverage of the neurocranium

## Abstract

**Background:**

Reconstruction of cranial composite defects, including all layers of the scalp and the neurocranium, poses an interdisciplinary challenge. Especially after multiple previous operations and/or radiation therapy, sufficient reconstruction is often only possible using microsurgical free flap transplantation. The aim of this study was to analyze the therapy of interdisciplinary cases with composite defects including the scalp and neurocranium.

**Methods:**

From 2009 to 2017, 23 patients with 18 free flaps and 10 pedicled/local flaps were analyzed. First choices for free flaps were muscle flaps followed by fasciocutaneous flaps.

**Results:**

Except for four patients, a stable coverage could be reached in the first operation. Three of these patients received a local scalp rotation flap in the first operation and needed an additional free flap because the local flap was no longer sufficient for coverage after wound healing deficiency or tumor relapse. The superficial temporal artery or external carotid artery served as recipient vessels. In special cases, venous grafts or an arteriovenous loop (AV loop) were used as extensions for the recipient vessels.

**Conclusions:**

In summary, an interdisciplinary approach with radical debridement of infected or necrotic tissue and the reconstruction of the dura mater are essential to reach a stable, long-lasting reconstructive result. Based on our experience, free flaps seem to be the first choice for patients after multiple previous operations and/or radiation therapy.

## Key Messages

- Defects, including all layers of the scalp and neurocranium, pose an interdisciplinary challenge
- Flap selection, recipient vessels, and outcome based on the current literature and our experience with microsurgical free tissue transfer were analyzed
- Especially after multiple previous operations and/or radiation therapy, sufficient reconstruction is often only possible using microsurgical free flap transplantation

## Background

Reconstruction of composite defects of the scalp and neurocranium represents a major challenge to both neurosurgeons and plastic and reconstructive surgeons, especially with regard to the inelastic surrounding tissue after previous operations or radiation therapy [1, 2]. Cranial composite defects can be caused by trauma, wound healing disorders, infection, burn injury, congenital lesions, tumor resection, osteomyelitis, or osteoradionecrosis [3].

The reconstruction of these composite defects includes the restoration of soft tissue, the protection of intracranial contents and, in selected cases, the restoration of the bony contour. The reconstructive goal is to generate a durable tissue cover that withstands trauma or radiation and heals quickly allowing necessary adjuvant treatments [4, 5]. The common approach for scalp reconstruction starts with a careful assessment of the patient and the potential defect, including the location, size, depth as well as the components of the defect [6].

In general, the simplest and most reliable method for reconstruction should be considered in all patients, but a number of patients exist in whom the defect size, the presence of infection, or previous radiation therapy and surgery make a more radical approach necessary [7]. Large defects may involve the entire thickness of the soft tissue, or even include calvarial bone and dura mater with cerebrospinal fluid leakage in patients with poor general performance [8, 9]. In those cases, chronic soft tissue infection or osteomyelitis often negatively influence the viability of the surrounding tissue and severely limit the use of locoregional flaps for reconstruction [10].

Despite great advances in tissue engineering, free tissue transfer remains as the only option for these cases allowing the preservation of the structural and functional status of the reconstructed area [11–17].

This article (1) outlines our approach for scalp reconstruction based on our experience with microsurgical free-tissue transfer for composite defects affecting the scalp and neurocranium; (2) describes a staging system for forehead and scalp defects; and (3) analyzes flap selection, recipient vessels, and outcome in a series of 23 consecutive patients.

## Methods

**Fig. 1:**
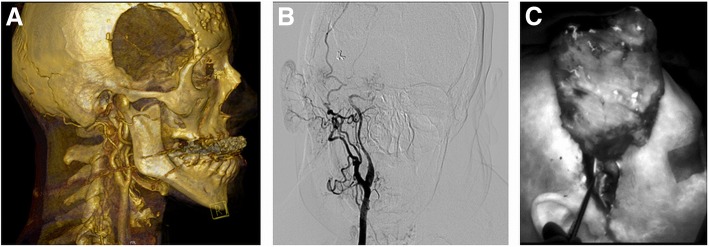
Preoperative imaging. CT angiography imaging with 3D reconstruction (**a**). Digital subtraction angiography of the head and neck vessels (**b**). Intraoperative fluorescence angiography using the SPY Elite Imaging System demonstrating excellent flap perfusion and the superficial temporal vessels (**c**)

Twenty three patients with composite defects of the neurocranium were operated between 2009 and 2017. The medical charts were reviewed to record the following data: patient characteristics [age, sex, etiology, defect size] and flap characteristics [flap components; recipient vessels]. Complications were collected and compared, as well as the functional outcome and follow-up period (at least more than 6 months). Before surgery, imaging of the defect, the surrounding tissue, and potential nutrition vessels was performed by digital subtraction angiography (DSA), MR imaging in combination with MRI angiography or CT angiography (Fig. 1). Preoperative imaging and the treatment plan were discussed in an interdisciplinary case presentation. Debridement and tumor resection were conducted both by the neuro- and plastic surgeon. Dura replacement was performed solely by the neurosurgeon. Defect reconstruction was performed by the plastic surgeon. The vascular surgeon created the arteriovenous loop.

Intraoperative fluorescence angiography (as described in the literature for example in [18]) by SPY Elite Imaging System (Novadaq Technologies Inc.) was performed during surgery using Indocyanin green to assess flap perfusion.

Additionally, a review of the literature was performed to define the options for cranial bone and soft tissue reconstruction with their advantages and disadvantages. Based on this review and our own experience, we present a treatment algorithm for the reconstruction of composite defects affecting the scalp and neurocranium. This study was approved by the ethical review committee of the Friedrich-Alexander-University of Erlangen-Nuremberg (AZ 169_15Bc).

## Results

Based on complete medical review, we performed a retrospective analysis of 23 patients who underwent 28 individually and interdisciplinary planned reconstructive treatments of scalp defects. Within the last 8 years, 18 free flaps and 10 pedicled/local flaps were performed. Most patients were female (61%) and mean age was 61 years (range 43–88 years).

The defects resulted from trauma or cancer treatment with most of the patients (16 of 23) having had multiple operations in the scalp area in other hospitals (Table 1). A broad range of malignancies caused the composite defects, including squamous cell carcinoma, glioblastoma, meningioma, adenocarcinoma, and angiosarcoma. In addition, most patients presented after radiation therapy, resulting in poor soft tissue quality and/or had a history of recurrent infections and/or osteomyelitis. Unstable scarring, delayed wound healing, and bone necrosis were noted in most of the patients in our series. Thirty percent of the patients pre-operatively suffered from liquor fistula (e.g., patient 2). Whenever possible, dura reconstruction with fascia lata or a part of the anterior rectus sheath was performed. Large defects of the dura mater were reconstructed with alloplastic material (equine collagen bio-matrix for dura regeneration, TissuDura®, Baxter, Unterschleißheim, Germany). Reconstruction of osseous defects was not performed in any case in our series.

**Table 1:** Patients included in the case series

Patient	Free flap	Pedicled flap	Flap	Recipient vessels	Indication for reconstruction	Gender	Age
1	1	–	Latissimus dorsi flap	Superior thyroid artery, external jugular vein	Chronic osteomyelitis, liquor fistula following meningioma resection	W	65
2	1	–	Rectus abdominis flap	Lingual artery, internal jugular vein	Infected seroma, liquorrhoe following meningioma resection and radiation	W	49
3	1	–	Latissimus dorsi flap	Lingual artery, internal jugular vein	Infected bone cement, wound healing disorder, chronic osteomyelitis following dermatofibrosarcoma protuberans resection	W	51
4	1 1	1	Rectus abdominis flap Latissimus dorsi flap Scalp rotation flap	External carotid artery, internal jugular vein external carotid artery, external jugular vein –	Subdural abscess, liquor fistula wound healing disorder following meningioma resection and radiation	M	67 68 69
5	–	1	Scalp rotation flap + Temporalis fascia flap + split skin graft	–	Epidural empyema following resection of breast cancer metastasis and radiation	W	58
6	1	–	Radial forearm flap	AV Loop (common carotid artery, internal jugular vein)	Wound healing disorder, infected bone cement following radiation and meningioma/neurofibroma resection	W	48
7	–	1	Pedicled trapezius flap + split skin graft	–	Meningioma resection hydrocephalus	W	81
8	1	–	Rectus abdominis flap	Superior thyroid artery, internal jugular vein	Liquor fistula, recurrent meningitis following glioblastoma resection and radiation	M	56
9	1	–	Radial forearm flap	Superficial temporal artery and vein (revision: external jugular vein + vein graft)	Infected bioglass prosthesis and wound healing deficit following craniocerebral injury	W	46
10	1	–	Rectus abdominis flap	External carotid artery, retromandibular vein	Recurrent abscess and wound healing disorder following meningioma resection	W	62
11	1	–	Latissimus dorsi flap	Superficial temporal artery and vein	Squamous cell carcinoma resection	M	81
12	1	–	Latissimus dorsi flap	Superficial temporal artery, retromandibular vein	Squamous cell carcinoma resection	M	74
13	1	1	Scalp rotation flap Latissimus dorsi flap	– superior thyroid artery, retromandibular vein (revision: lingual artery)	Wound healing disorder following adenoid cystic carcinoma resection tumor recurrence	W	45 46
14	–	1	Juri flap + split skin graft	–	Squamous cell carcinoma	M	74
15	1	–	Latissimus dorsi flap	Superficial temporal artery and vein	Wound healing disorder following glioblastoma resection and radiation	W	47
16	1	–	Latissimus dorsi flap	Superficial temporal artery and vein	Liquor fistula, wound healing disorder following glioblastoma resection and radiation	W	43
17	1	1	Scalp rotation flap Latissimus dorsi flap	– Superficial temporal artery and vein	Liquor fistula and wound healing disorder following meningioma resection and radiation	M	44 46
18	1	–	Seratus anterior flap	Superficial temporal artery and vein	Wound healing disorder and liquor fistula following aneurysm surgery	M	59
19	1	–	Latissimus dorsi flap	Superficial temporal artery and vein	Skin necrosis, infection, exposed shunt system following trauma	W	68
20	1	1	Scalp rotation flap Latissimus dorsi flap	Superficial temporal artery and vein	Recurrent abscesses and wound healing disorder following meningioma resection	M	65
21	–	1	Scalp rotation flap	–	Abscess, exposed bone/osteosynthesis material following astrocytoma resection	W	61
22	–	1	Scalp rotation flap	–	Angiosarcoma, radiation	M	88
23	–	1	Scalp rotation flap	–	Glioblastoma, radiation	W	69

**Fig. 2:**
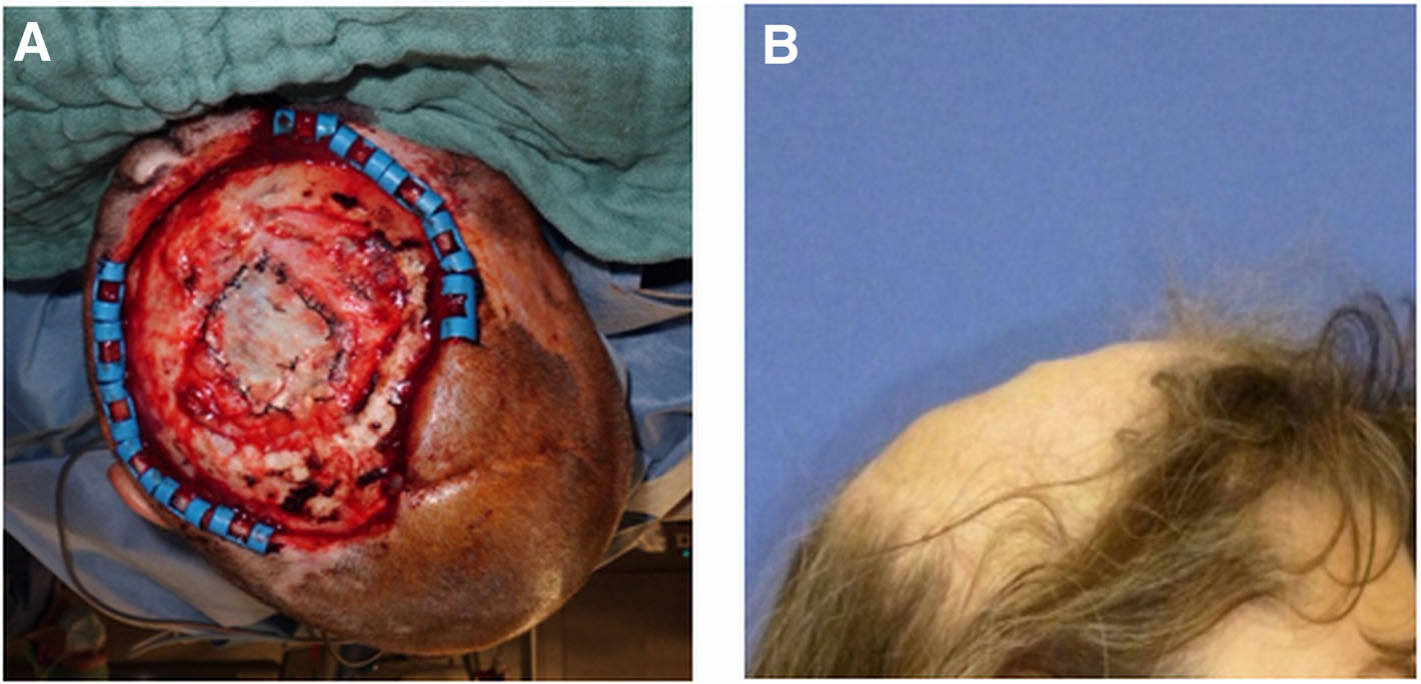
Clinical case (patient 13): composite defect of the cranium after recurrence of an adenoid cystic carcinoma. Intraoperative situs after tumor resection and dura replacement (**a**). Clinical aspect 2 years after reconstruction with a latissimus dorsi free flap (recipient vessels: superior thyroid artery and retromandibular vein) (**b**)

**Fig. 3:**
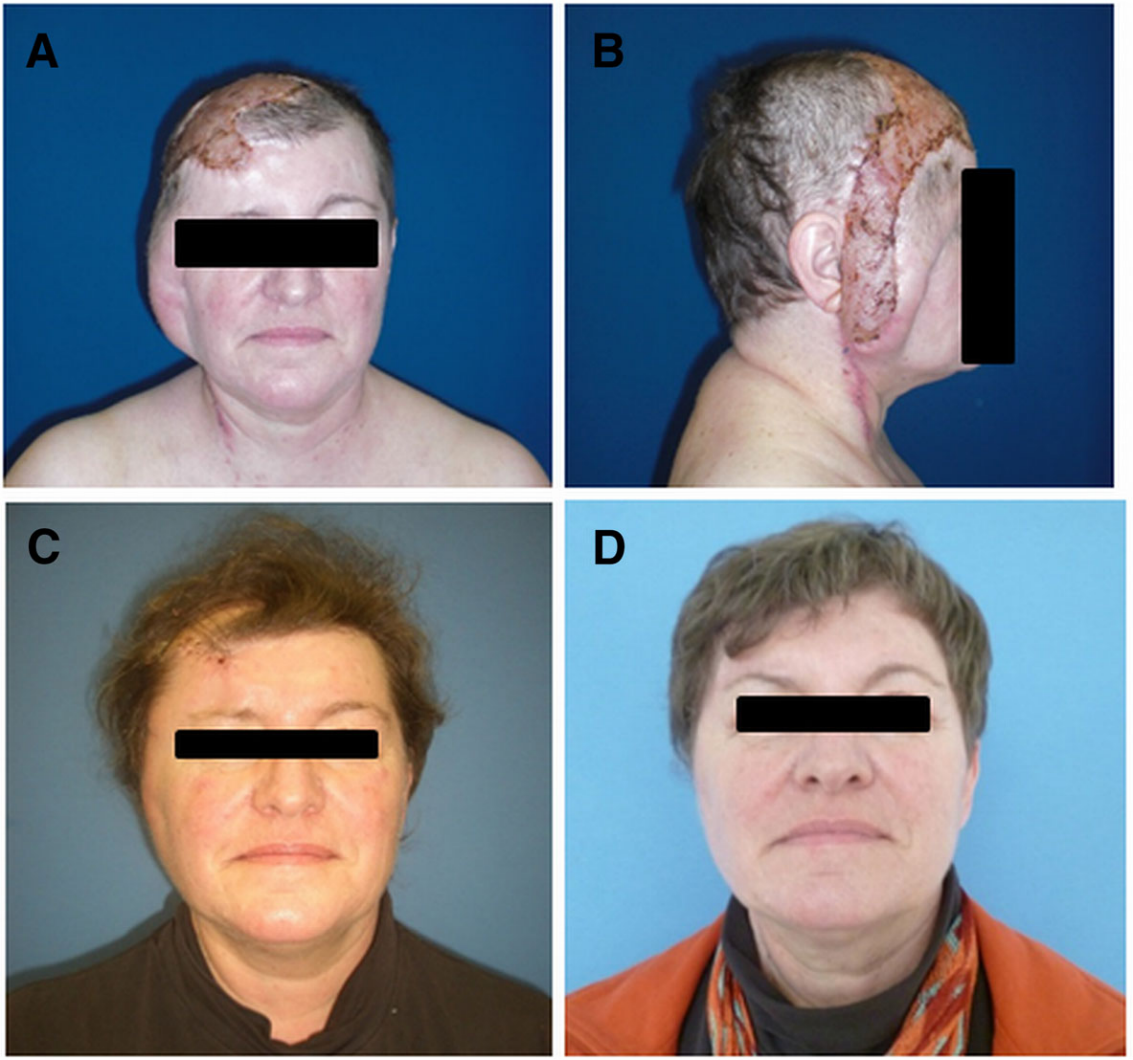
Clinical case (patient 3): chronic infected composite defect after several operations because of dermatofibrosarcoma protuberans and cranioplasty. 3 weeks after transplantation of a latissimus dorsi free flap (recipient vessels: lingual artery/internal jugular vein) (**a**, **b**). 4 weeks after partial excision of the latissimus flap and forehead lift (**c**). Final aspect after brow lift and blepharoplasty (**d**)

**Fig. 4:**
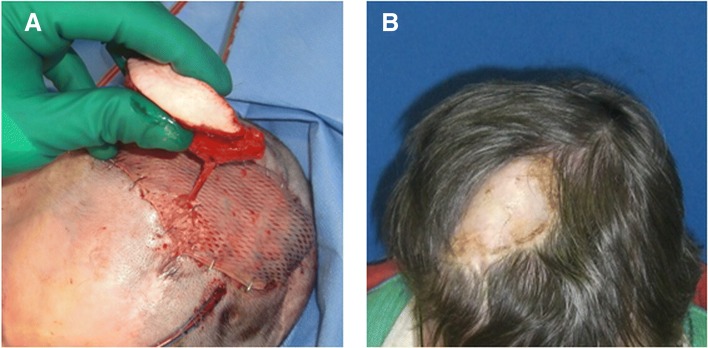
Clinical case (patient 10): recurrent abscess and wound healing disorder following meningioma resection. Intraoperative situs demonstrating the rectus abdominis free flap with a perforator-based monitor island (recipient vessels: external carotid artery/retromandibular vein) (**a**). Clinical aspect approximately 6 months later (**b**)

Free flaps (*n* = 18) were first choice for defect reconstruction including the latissimus dorsi flap (*n* = 11), rectus abdominis flap (*n* = 4), serratus anterior flap (*n* = 1), and radial forearm flap (*n* = 2) (Table 2). Free muscle flaps (*n* = 16) were chosen for deep defects, chronic wounds associated with infection (e.g., osteomyelitis) or irradiation (Figs. 2, 3 and 4). In these cases, secondary correction operations were routinely offered to the patients. One relatively young female patient underwent a number of secondary cosmetic improvements such as forehead brow lift and upper-lid blepharoplasty (Fig. 3).

**Table 2:** Flap statistics/recipient vessels

Flaps	Number	Type	Number
Free flaps			
Latissimus dorsi	11	Muscle	16
Rectus abdominis	4		
Serratus anterior	1		
Radial forearm	2	Fasciocutaneous	2
Local flaps			
Trapecius flap and split thickness skin graft	1	Muscle	1
Juri flap + split skin graft	1	Fasciocutaneous	10
Scalp rotation flap + temporalis fascia flap + split skin graft	1		
Scalp rotation flap	8		
Recipient vessels	Number	Type	Number
Superficial temporal artery	9	Head	9
Superior thyroid artery	3	Neck	10
Lingual artery	3		
External carotid artery	3 (2 interposition of vein grafts)		
AV Loop (common carotid artery)	1		
Superficial temporal vein	8	Head	8
External jugular vein	3 (1 interposition of vein graft)	Neck	11
Internal jugular vein	5		
Retromandibular vein	3

When using muscle flaps for coverage, perforator-based monitor islands were used whenever possible [19]. The perforator island was raised only on a single perforator, and the underlying muscle tissue was completely covered by split-thickness skin grafts during the first operation. This technique offers the advantage that the monitor island can be removed bedside, and no second split-skin grafting procedure is necessary (Fig. 4). In two cases, a radial forearm flap (=fasciocutaneous flap) was used for defect reconstruction.

No free flap loss was registered in our series, but emergency revision of free flaps was necessary in two patients. In one patient, the flap anastomosis had to be revised due to a kinking of the pedicle vessel and/or the donor vessel (superior thyroid artery). In the second case, insufficient venous drainage was the reason for revision. In the first operation, the radial forearm flap was anastomosed to the superficial temporal vessels. In the first night, venous congestion occurred and the patient was returned to the operating room. Venous drainage was improved by connecting the cephalic vein with an interposed vein graft to the external jugular vein. Except for four patients, a stable coverage could be achieved in the first operation. In one patient, a second free flap was necessary because of a relapse of adenoid cystic carcinoma (Fig. 2). First, a scalp rotation flap and readvancement of a prior transplanted latissimus dorsi flap was performed. 1 year later, MR imaging revealed tumor recurrence. Followed by a neurosurgical resection and dura mater reconstruction, the defect was covered by a latissimus dorsi free flap. The second patient received a scalp rotation flap in the first operation because of recurrent abscess after meningioma resection. Because of wound healing disorder and recurrent fistula, an additional latissimus dorsi free flap was performed for stable coverage.

The third patient received two sequential free flaps and one local flap. The composite defect resulted from multiple previous operations, radiation therapy as well as a chronic wound situation with liquor fistula and subdural abscess after meningioma resection. First, a rectus abdominis free flap was performed. Because of unstable coverage, recurrent ulcer and a secondary defect a latissimus dorsi free flap was necessary 1 year later. In consequence of subdural abscess and unstable coverage 1 year after revision surgery, a scalp rotation flap was performed which finally solved the problem. The fourth patient suffered from wound healing disorder and liquor fistula following meningioma resection and radiation therapy. In the first operation, dura repair and a scalp rotation flap were performed. Because of recurrent wound healing disorder, a latissimus dorsi free flap was necessary.

In most cases, we performed CT angiography (14/18) to identify the recipient vessels. The superficial temporal artery as well as the external carotid artery with its cervical branches, served as first choice for recipient vessels. In three cases venous grafts were used as extensions for the recipient vessels to place the flaps accurately (Table 2). In one case, an arteriovenous loop (AV loop) was created between the common carotid artery and the internal jugular vein. This patient suffered from wound healing disorder after radiation therapy and several operations to excise neurofibromas and meningiomas. Infected bone cement, from former operations, was exposed. Because of the patient’s habitude, the reduced health condition and the defect location a radial forearm flap connected with an AV loop was performed. The postoperative course was uneventful and a stable coverage achieved.

## Discussion

The defect depth and size, as well as the simultaneous consideration of the patient’s general condition should be taken into account for scalp reconstruction [20, 21]. A detailed understanding of scalp anatomy and perfusion, as well as the quality of the soft tissue, is critical for sufficient reconstruction [22, 23]. It should be distinguished between small (< 10 cm^2^) and moderately (10–50 cm^2^) sized defects in patients with good general health where full closure is achieved easily and esthetic aspects (e.g., eyebrow symmetry, hairline, avoidance of alopecia) are the challenge in those cases [6] and large defects (> 50 cm^2^) in patients with poor general performance status where complete per se closure is the primary goal [3, 24].

**Fig. 5:**
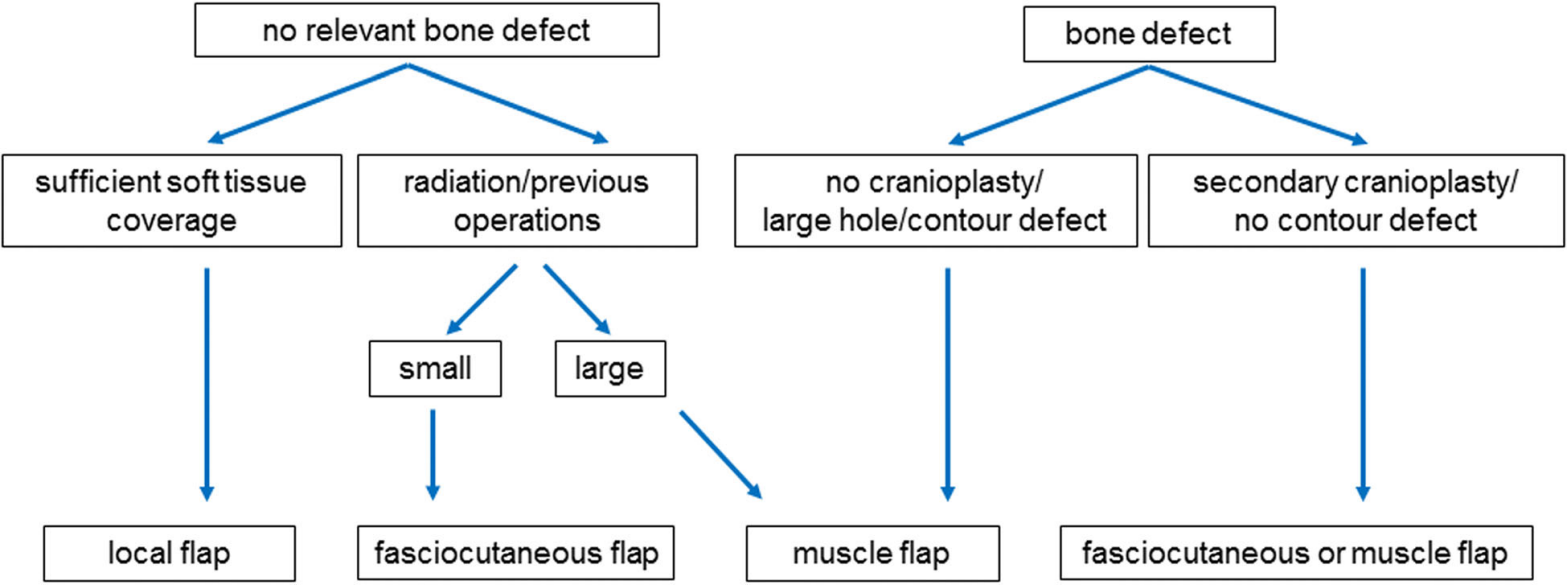
Flow process chart: the reconstructive approach is dependent on the defect components, size as well as the quality of the surrounding soft tissue

According to the results of our case series and in light of the current literature, a flow process chart was developed (Fig. 5) [6].

The first point to be addressed is whether there is a relevant osseous defect requiring cranioplasty or not. On the other hand, one has to bear in mind that cranioplasty is associated with a high complication rate (up to 30%) [25]. The main complications are bone resorption and infection leading to revision surgery [26, 27]. This issue should be discussed with the neurosurgeon against the background of the following considerations: protection against trauma, restoration of appearance, and the putative “syndrome of the trephined.” [28] So far, there is no consensus about the defect size that requires cranioplasty [29]. Young and active patients may require a protective cranioplasty, whereas older and inactive patients may have only a limited risk of injury. The osseous defect leads to a depression of the skin and a malformed appearance. The defect size and location are important aspects, because lesions in the frontal area anterior to the hairline are more obvious than at the rest of the cranium [30]. The “syndrome of the trephined” or “syndrome of the sunken skin flap” includes symptoms such as dizziness, fatigability, vague discomfort, mental depression, and intolerance to vibration [28]. There is the possibility of an improvement in neurological function after cranioplasty that may be related to changes in cerebrospinal fluid circulation [31]. The literature describes how simultaneous free flap scalp reconstruction and cranioplasty can be combined without increasing complications, even with multiple risk factors in uninfected situations [32].

**Fig. 6:**
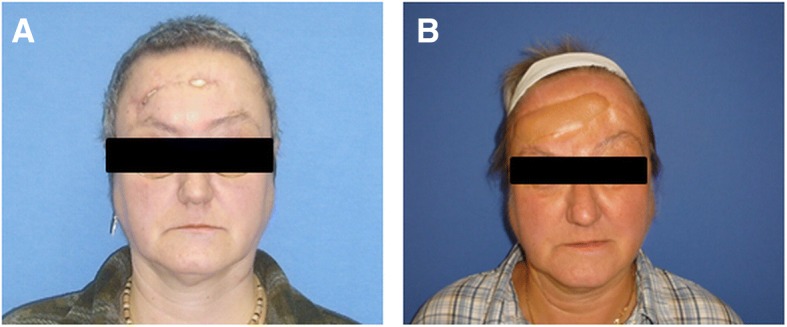
Clinical case (patient 9): chronic infected wound healing disorder following craniocerebral trauma and reconstruction of the cranial vault with a bioglass CAD model (**a**). Clinical aspect approximately 3 years later after reconstruction with a radial forearm flap (**b**)

In our own collective, most patients were rather elderly and inactive, coming along with a lower risk for skull injury. Furthermore, most defects were located above the hat brim line so that esthetic considerations were not as important as in frontal defects. Only in one patient the defect was adjacent to the hairline. Because there was no significant impression of the scalp, inconspicuous appearance was achieved with a radial forearm flap (Fig. 6).

In our case series, most patients required scalp reconstruction because of chronic infected wounds after previous multiple surgical interventions, radiation therapy, and/or prior alloplastic cranioplasty. Besides the fact that cranioplasty is associated with a high complication rate, we believe that the above mentioned patient characteristics are strong contraindications for cranioplasty [25, 33, 34].

The quality of soft tissue coverage is the second point that should be addressed. The simplest possible method of reconstruction should be considered in all patients, while ensuring adequate resection, radical debridement, and a good functional result. Standard methods for scalp reconstruction include local flaps with and without previous skin expansion, skin grafts, and free flaps. If small (< 10 cm^2^) to moderately sized defects (10–50 cm^2^) in healthy patients involve only skin, esthetic aspects (e.g. eyebrow symmetry, hairline, avoidance of alopecia) are the main challenge [35]. If the periosteum is intact and/or the defect leaves a well-perfused wound ground, a skin graft may be adequate to cover the defect [1, 31]. On the other hand, postoperative radiation is not well tolerated, and the esthetic outcome is inferior compared to other methods [1, 36].

If there is sufficient soft tissue near the defect, a local flap can be designed. Scalp flaps offer the advantage of replacing the defect with well-perfused and hair-bearing tissue. But their relative inelasticity limits the flap size resulting in additional skin grafting of the donor site [4]. In this regard tissue expansion can be a useful technique. If a quick reconstruction is necessary or in case of infected wound situation tissue expansion is not possible. [37, 38].

Local flaps, based on the rich vascular supply, are sufficient to cover moderate (10–50 cm^2^) and large (> 50 cm^2^), full-thickness defects of the scalp. On the other hand, the use of local flaps might not be appropriate after multiple previous surgery and/or radiation, resulting in decreased vascularization [20, 39–41].

In case of severe infection associated with acute or chronic osteomyelitis, local recruitment of traumatized or insufficiently perfused tissue imposes a high risk of flap failure [2]. In our case series, two patients received a local scalp rotation flap in the first operation and needed an additional free flap for stable coverage because of wound healing disorder. In both cases, the patients had multiple operations prior reconstruction, radiation therapy, and/or chronic soft tissue infection. The use of well-perfused tissue has been generally accepted to promote wound healing and to treat infected chronic wounds [42]. In the event of defects involving the calvarial bone, free flaps should be considered in patients with good general health as well as in elder multimorbid patients [8, 43]. The disadvantages of free flaps, including the duration of the surgical procedure, donor side morbidity, the risk of flap loss, and the lack of hair growth in the reconstructed area should be carefully discussed.

In our case series, imaging of the head and neck vessels was performed using different methods. Digital subtraction angiography (DSA) of the head and neck vessels is one option to determine the nutrition vessels for the planned flap. This method offers the advantage of percutaneous transluminal angioplasty of stenotic neck vessels by the radiologist. MR imaging can be combined with angiography and provides a good overview of the surrounding soft tissue and the recipient vessels. MRI angiography may be of less resolution than DSA or CT angiography, but reduces the amount of radiation exposure. CT angiography is a reliable method with a sufficient resolution and in our opinion the gold standard for the preoperative imaging of scalp composite defects and the recipient vessels [44].

The pathological features of the scalp lesion are crucial for choosing the appropriate reconstruction method [6, 32]. If there is a contour defect with a large sunken hole and no cranioplasty is planned, a muscle flap is the adequate choice in our opinion. In cases in which a secondary cranioplasty is planned, or if there is no contour defect, it has to be discussed individually with the patient and neurosurgeon which option (fasciocutaneous or muscle flap) fits best in the individual situation. Free muscle flaps covered with skin grafts and free muscle flaps with a skin paddle seem to be equally reliable and durable [1, 32]. Free tissue transfer remains a mainstay for the treatment of scalp defects, with latissimus dorsi-based flaps, demonstrating excellent versatility for a broad range of defects [45, 46]. In the literature, calvarial bone reconstruction using a chimeric latissimus dorsi and serratus anterior and rib free flap has been described [47]. In cases with cerebrospinal fluid leakage, dural healing can be promoted by well-vascularized, healthy tissue from a distant site [31]. The creation of an arteriovenous (AV) loop is a helpful tool for patients lacking sufficient recipient vessels [48]. Although controversially discussed, we prefer a two-staged regimen with the creation of the AV loop first and the reconstruction procedure after 1–2 weeks [49]. From our point of view, a two-staged procedure minimizes flap loss due to vascular complications concerning the AV loop such as secondary hemorrhage, insufficient blood flow, or thrombosis [50].

Patients receiving free tissue transfer displayed a mean age of 58 years (range 43–81). Patients treated with free tissue transfer were younger compared to patients treated with local tissue transfer (mean age 64; range: 44–88). There is evidence that the medical complication rate increases with patient’s age [51]. But from our point of view, the patient age is not the determining variable in choosing the reconstructive procedure. If a rigorous assessment is performed prior to surgery and the patients are treated in a center with microsurgical expertise, we believe that free tissue transfer is a safe and reliable procedure even though for elderly patients [21, 43, 51].

## Conclusion

In summary, a careful assessment and discussion of these complex cases in an interdisciplinary team (plastic surgeons, vascular surgeons, neurosurgeons, radiologists, and oncologists) is indispensable for a long-lasting treatment success. Moreover, free tissue transplantation is a reliable and safe procedure for reconstruction of large scalp or forehead defects after traumatic injury, tumor resection, multiple previous operations, failed local flaps, radiation therapy, and chronic infected wounds. In our hands, free flaps are often a better option than an insufficient coverage by skin grafts or local flaps. Sufficient recipient vessels are essential, and the superficial temporal vessels as well as the external carotid artery with its branches represent a safe choice. In special cases, venous grafts or AV loops should be used as extensions to reach an optimal flap positioning and stable wound healing.
